# Nutraceutical Potential of Tinctures from Fruits, Green Husks, and Leaves of *Juglans regia* L.

**DOI:** 10.1155/2014/501392

**Published:** 2014-01-28

**Authors:** Urszula Gawlik-Dziki, Agata Durak, Łukasz Pecio, Iwona Kowalska

**Affiliations:** ^1^Department of Biochemistry and Food Chemistry, University of Life Sciences, Skromna Street 8, 20-704 Lublin, Poland; ^2^Department of Biochemistry and Crop Quality, Institute of Soil Science and Plant Cultivation, State Research Institute, Czartoryskich Street 8, 24-100 Pulawy, Poland

## Abstract

The aim of this study was to assess the phenolic composition and nutraceutical potential of tinctures from fruits in two stages of maturity (F3, younger; F25, older), green husks (GH), and leaves (L) of *Juglans regia* L. In all extracts gallic, protocatechuic, 3-caffeoylquinic, 3-**ρ**-coumaroylquinic, 4-caffeoylquinic, 4-**ρ**-coumaroylquinic, and **ρ**-coumaric acids and quercetin-3-*O*-deoxyhexoside were detected using UPLC-MS technique. Caffeic acid hexoside I and quercetin-3-*O*-deoxyhexoside I have been identified in GH tincture. The highest ability to chelate Fe^2+^ was observed for GH tincture (EC_50_ = 71.01 ± 3.55 mg FM/mL), whereas the lowest was observed (EC_50_ = 131.06 ± 6.55 mg FM/mL) for F3 tincture. The highest reducing power was found for F3 and F25 (EC_50_ = 32.47 ± 1.53 and 36.07 ± 1.72 mg FM/mL, resp.). Ability of tinctures to prevent lipids against oxidation was relatively low. The highest activity (EC_50_ = 126.49 ± 6.32 mg FM/mL) was determined for F25. Tested tinctures showed relatively high antiradical activity—EC_50_ values ranged from 100.56 ± 5.03 to 129.04 ± 6.45 mg FM/mL for L and F25, respectively. The results obtained suggest that *J. regia* can be a source of bioactive compounds with antioxidant properties.

## 1. Introduction

The genus *Juglans *(family Juglandaceae) comprises several species and is widely distributed throughout the world. Green walnuts, shells, kernels and seeds, bark, and leaves are used in the pharmaceutical and cosmetic industries [1, 2]. Walnut's green husk is a by-product of the walnut production, having scarce use. Thus, using husk as a source of phytochemicals will increase the value of the walnut production as well as offer utilization for a by-product, which is produced in a large quantity [3].

Different works demonstrated the potential antioxidant of walnut products, (mainly fruits, but also leaves) [1] and liqueurs produced by green fruits [2]. In addition to antioxidant activity, several studies have demonstrated the antimicrobial activity of phenols and/or phenolic extracts of *Juglans regia* [4], making them a good alternative to antibiotics and chemical preservatives.

Nowadays, there is an increasing interest in the substitution of synthetic food antioxidants by natural ones. The antioxidant compounds from waste products of food industry could be used for protecting the oxidative damage in living systems by scavenging oxygen free radicals and also for increasing the stability of foods by preventing lipid peroxidation [5]. Special attention is focused on their extraction from inexpensive or residual sources coming from agricultural industries, such as walnut fruit. Furthermore, leaves are easily available in abundant amounts. Walnut leaves are considered to be a source of healthcare compounds and have been intensively used in traditional medicine for the treatment of venous insufficiency, hemorrhoids, hypoglycemia, diarrhea, and fungal or microbial infections. Dry walnut leaves are also frequently used as infusions [1]. The shell is used as a filtration media to separate crude oil from water [6] and the walnut green husk is the basic material for the traditional walnut liqueur [2]. The results obtained by Oliveira et al. [3] and Carvalho et al. [7] showed the potential of this low cost natural material as source of phenolic compounds with antiradical and antimicrobial activities. Phenolic compounds play a number of crucial roles in the complex metabolism of plants and also of fruit trees. They are involved in physiological processes of fruit tree growth and development and affect different aspects of fruit pre- and postharvest life [8].

Foods of plant origin, such as fruits and vegetables, and whole grain products have been suggested as a natural source for antioxidants. Antioxidants can play an important role in disease prevention and health maintenance. Plant-derived products can be used either as a source of antioxidants in industry or for medicinal purposes. The antioxidant effect shown by these products proceeds from phenolic compounds and phytochemicals, which is protected from harmful effects of free radicals. Walnut possesses a high content of *α*-tocopherol, a vitamin E family compound, which has antioxidant activity, mainly in the prevention of lipid oxidation process [1].

The aim of this study was to assess the nutraceutical potential of *J. regia *fruits, green husks, and leaves ethanolic extracts. For this purpose, we have made a qualitative assessment of phenolic compounds. Furthermore, we analyzed the total phenolic content, phenolic acids, and flavonoids in extracts, their antioxidant activity, and their effect on the activity of some enzymes from the class of oxidoreductases.

## 2. Materials and Methods 

### 2.1. Chemicals

ABTS (2,2′-azino-bis(3-ethylbenzothiazoline-6-sulphonic acid)), Folin-Ciocalteau reagent, Arnov reagent, gallic acid, quercetin, ferrozine, lipoxygenase, xanthine oxidase, catalase, and linoleic acid were purchased from Sigma-Aldrich company (Poznan, Poland). Acetonitrile and methanol gradient HPLC grade and formic acid LC-MS grade for LC-UV-MS separations were purchased from J.T. Baker (Phillipsburg, NJ). Water was purified in-house with a Milli-Q water purification system Simplicity-185 (Millipore Co.). All other chemicals were of analytical grade.

### 2.2. Preparation of Samples

The experimental material consisted of walnut fruits harvested at two stages of maturity (July 3, 2012 and July 25, 2012). Green husks and leaves were harvested on September 23, 2012. The research material was obtained from a private plantation of walnut in Radzyń Podlaski, Poland. The immature fruit, green husks, and dried leaves were shredded and were used to obtain extracts. For alcohol extraction 5 g of each plant material was extracted in 50 mL of EtOH (70% v/v) for 2 weeks in darkness at 25°C, and then the supernatant was recovered. Obtained extracts as follows:

 F3, extract from walnut fruits harvested 3.07.2012,
 F25, extract from walnut fruits harvested 25.07.2012,
 GH, extract from green husks of walnut,
 L, extract from walnut leaves.

The final extracts concentration was 0.1 g fresh mass (FM)/mL.

### 2.3. Ultraperformance Liquid Chromatography

Compounds of interest were analyzed using a Waters ACQUITY UPLC system (Waters Corp., Milford, MA, USA), consisting of a binary pump system, sample manager, column manager, and PDA detector (also from Waters Corp.). Waters MassLynx software v.4.1 was used for acquisition and data processing. The samples were separated on a BEH C18 column (100 mm × 2.1 mm i.d., 1.7 *μ*m, Waters Corp., Milford, MA, USA), which was maintained at 40°C. The flow rate was adjusted to 0.40 mL/min. The following solvent system: mobile phase A (0.1% formic acid in Milli-Q water, v/v) and mobile phase B (0.1% formic acid in MeCN, v/v), was applied. The gradient program was as follows: 0-1.0 min, 5% B; 1.0–24.0 min, 5–50% B; 24.0-25.0 min, 50–95% B; 25.0–27.0 min, 95% B; 27.0–27.1 min, 95-5% B; 27.1–30.0 min, 5% B. Samples were kept at 8°C in the sample manager. The injection volume of the sample was 2.0 *μ*L (full loop mode). Strong needle wash solution (95 : 5, methanol-water, v/v) and weak needle wash solution (5 : 95, acetonitrile-water, v/v) were used. UV-PDA data was acquired from 220 nm to 480 nm, at 5 point/s rate, 3.6 nm resolution. The separation was completed in 30 minutes. Peaks were assigned on the basis of their UV spectra, mass to charge ratio (*m/z*) and ESI-MS/MS fragmentation patterns.

The MS analyses were carried out on a TQD mass spectrometer (Waters Corp.) equipped with a Z-spray electrospray interface. The following instrumental parameters were used for ESI-MS analysis of phenolic compounds (negative ionization mode): capillary voltage, 2.8 kV; cone voltage, 40 V; desolvation gas, N_2_ 800 L/h; cone gas, N_2_ 100 L/h; source temp. 140°C, desolvation temp. 350°C. Compounds were analyzed in full scan mode (mass range of 100–1600 amu was scanned).

For ESI-MS/MS, selected ions were fragmented using a collision energy of 15 V (phenolic acids derivatives) or 25 V (flavonoids derivatives) and collision gas (argon) at 0.1 mL/min.

### 2.4. Phytochemical Analysis

Total phenols were estimated according to the Folin-Ciocalteau method [9]. A 0.1 mL sample of the extract was mixed with 0.1 mL of H_2_O, with 0.4 mL of Folin reagent (1 : 5 H_2_O), and after 3 min with 2 mL of 10% Na_2_CO_3_. After 30 min, the absorbance of mixed samples was measured at a wavelength of 720 nm. The amount of total phenolics was expressed as gallic acid equivalents (GAE).

Total flavonoids were estimated according to Lamaison method [10]. A 1 mL sample of the extract was mixed with 1 mL AlCl_3_ × 6H_2_O (2% v/v) and after 10 min the absorbance of mixed samples was measured at a wavelength of 430 nm. The amount of flavonoids was expressed as quercetin acid equivalents (QE).

Total phenolic acids were determined according Arnov method [11]. 1 mL of distilled water was mixed with 0.2 mL of extract, 0.2 mL HCl (0.5% v/v), 0.2 mL of Arnova reagent, and 0.2 mL NaOH (1 M). The absorbance of mixed samples was measured at a wavelength of 490 nm. The amount of phenolic acids was expressed as caffeic acid equivalents (CAE).

### 2.5. Determination of Iron Chelating Activity

The method of Decker and Welch [12] was used to investigate the ferrous ion chelating ability of proteins and hydrolysates. Briefly, the sample (0.5 mL) was added to 0.2 mL 2 mM FeCl_2_ solution and 0.2 mL 5 mM ferrozine. The mixture was shaken vigorously and incubated at room temperature for 10 min. The absorbance was subsequently measured at 562 nm in the spectrophotometer. The percentage of inhibition of ferrozine-Fe^2+^ complex formation was given according to the formula:

(1) $$\begin{array}{c} \%\;\text{chelation}\;\;\text{activity}=\left[1-\left(\frac{A_{s}}{A_{c}}\right)\right]\times 100, \end{array}$$

where *A*_*s*_ is absorbance of sample and *A*_*c*_ is absorbance of control.

All assays were performed in triplicate.

### 2.6. Determination of Reducing Power

Reducing power was determined by the method of Oyaizu [13]. A 0.5 mL of extract was mixed with 0.5 mL (200 mM) of sodium phosphate buffer (pH 6.6) and 0.5 mL potassium ferricyanide (1% v/v) and samples were incubated by 20 min at 50°C. After that, 0.5 mL of TCA (10% v/v) was added and samples were centrifuged at 650 g by 10 min. Upper layer (1 mL) of supernatant was mixed with 1 mL of distilled water and 0.2 mL of ferric chloride (0.1% v/v). The absorbance was subsequently measured at 700 nm in the spectrophotometer.

### 2.7. Inhibition of Linoleic Acid Peroxidation [14]

Ten microliters of sample was added into a test tube together with 0.37 mL of 0.05 M phosphate buffer (pH 7.0) containing 0.05% Tween 20 and 4 mM linoleic acid and then equilibrated at 37°C for 3 min. The peroxidation of linoleic acid in the above reaction mixture was initiated by adding 20 *μ*L of 0.035% hemoglobin (in water), followed by incubation at the same temperature in a shaking bath for 10 min and stopped by adding 5 mL of 0.6% HCl (in ethanol). The hydroperoxide formed was assayed according to a ferric thiocyanate method with mixing in order of 0.02 M ferrous chloride (0.1 mL) and 30% ammonium thiocyanate (0.1 mL). The absorbance at 480 nm (*A*_*s*_) was measured with a spectrophotometer for 5 min. The absorbance of blank (*A*_0_) was obtained without adding hemoglobin to the above reaction mixture; the absorbance of control (*A*_100_) was obtained with no sample addition to the above mixture. Thus, the antioxidative activity of the sample was calculated as

(2) $$\begin{array}{c} \%\;\text{inhibition}=1-\left[\frac{\left(A_{s}-A_{0}\right)}{\left(A_{100}-A_{0}\right)}\right]\times 100\%. \end{array}$$

### 2.8. Determination of ABTS Radical Scavenging Activity

Free radical-scavenging activity was determined by the ABTS^•+^ method according to [15]. This reaction is based on decolourization of the green colour of the free ABTS radical cation (ABTS^•+^). The radical solution was prepared with ABTS and potassium persulfate, diluted in ethanol, at final concentration of 2.45 mM, and left at dark for 16 h to allow radical development. The solution was diluted to reach absorbance measures around 0.70–0.72 at 734 nm. 1.8 mL ABTS^•+^ solution was mixed with 0.04 mL of each sample. The absorbance was measured after one minute of reaction at 734 nm. 70% ethanol was used as blank. Percentage inhibition of the ABTS^•+^ radical was then calculated using the following equation:

(3) $$\begin{array}{c} \text{Scavenging}\;\%=\left[1-\left(\frac{A_{s}}{A_{c}}\right)\right]\times 100, \end{array}$$

where *A*_*s*_ is absorbance of sample and *A*_*c*_ is absorbance of control (ABTS solution).

All assays were performed in triplicate.

Antioxidant activities (except reducing power) were determined as EC_50_—extract concentration (mg FM/mL)—provided 50% of activity based on a dose-dependent mode of action. In the case of reducing power EC_50_ is the effective concentration at which the absorbance was 0.5 and was obtained by interpolation from linear regression analysis.

### 2.9. Effect on the Activity of Some Enzymes from the Class of Oxidoreductases

Inhibition of lipoxygenase (LOXI) activity was determined spectrophotometrically at a temperature of 25°C by measuring the increase of absorbance at 234 nm over a 2 min period [16]. The reaction mixture contained 2.45 mL of 1/15 mol/L phosphate buffer, 0.02 mL of lipoxygenase solution (167 U/mL), and 0.05 mL of inhibitor (*Juglans regia* extract) solution. After preincubation of the mixture at 30°C for 10 min, the reaction was initiated by adding 0.08 mL 2.5 mmol/L linoleic acid. One unit of LOX activity was defined as an increase in absorbance of 0.001 per minute at 234 nm. 0.08 mL 2.5 mmol/L linoleic acid. One unit of LOX activity was defined as an increase in absorbance of 0.001 per minute at 234 nm.

The inhibition of xanthine oxidase (XOI) activities with xanthine as a substrate was measured spectrophotometrically [17], with the following modification: the assay mixture consisted of 0.5 mL of test solution, 1.3 mL of 1/15 mol/L phosphate buffer (pH 7.5), and 0.2 mL of enzyme solution (0.01 U/mL in 1/15 M phosphate buffer). After preincubation of the mixture at 30°C for 10 min, the reaction was initiated by adding 1.5 mL of 0.15 mmol/L xanthine solution. The assay mixture was incubated at 30°C and the absorbance (295 nm) was measured every minute for 10 min. XO activity was expressed as the percentage inhibition of XO in the above assay mixture system and was calculated as follows:

(4) $$\begin{array}{c} \%\;\text{inhibition}=\left(\frac{1-\Delta A/\min_{\text{test}}}{\Delta A\;\min_{\text{blank}}}\right)\times 100, \end{array}$$

where Δ*A*/min⁡_test_ is the linear change in absorbance per minute of test material and Δ*A*min⁡_blank_ is the linear change in absorbance per minute of blank.

Influence on catalase (CAT) activity was assayed by the method of Claiborne [18] with some modification. The assay mixture consisted of 1.85 mL phosphate buffer (0.05 M, pH 7.0), 1.0 mL H_2_O_2_ (0.019 M), 0.1 mL of test solution, and 0.05 mL of enzyme solution (60 U/mL). The decomposition of H_2_O_2_ was determined directly by the extinction at 240 nm per unit time (3 min), which was used as a measure of catalase activity. The catalase activity was expressed as l mol of H_2_O_2_ consumed per min.

### 2.10. Statistical Analysis

All tests were conducted in triplicate. All experimental results were mean ± S.D. of three parallel measurements and data were evaluated by using one-way analysis of variance. The statistical differences between the treatment groups were estimated through Tukey's test. Statistical tests were evaluated by using the Statistica 6.0 software (StatSoft, Inc., Tulsa, USA). All the statistical tests were carried out at a significance level of *α* = 0.05.

## 3. Results and Discussion

### 3.1. Identification of Phenolic Compounds

**Figure 1:**
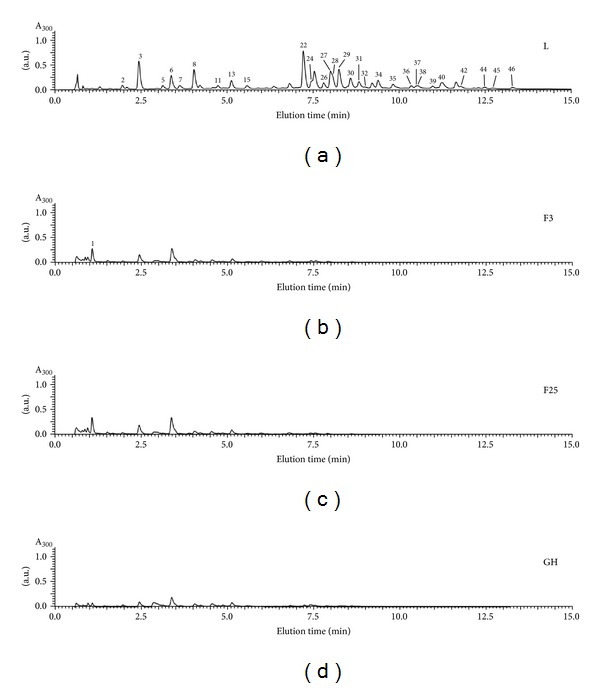
UPLC chromatograms of tinctures from different parts of *J. regia*. L: extract from walnut leaves, F3: extract from walnut harvested 3.07.2012, F25: extract from walnut harvested 25.07.2012, and GH: extract from green husks of walnut.

In all extracts prepared, the following eight phenolic compounds were detected: gallic, protocatechuic, 3-caffeoylquinic, 3-**ρ**-coumaroylquinic, 4-caffeoylquinic, 4-**ρ**-coumaroylquinic, and **ρ**-coumaric acids, as well as quercetin-3-*O*-deoxyhexoside. Furthermore, ethanolic extract of leaves is characterized by the greatest diversity of phenolic compounds from the tested samples, as presented in Table 1 and Figure 1.

On the other hand, two phenolic compounds that have been identified in the extract of the green husks were caffeic acid hexoside I and quercetin-3-*O*-deoxyhexoside I. These compounds, in turn, do not appear in the extracts of the fruits in both stages of maturity. Unfortunately, we were unable to identify the dominant compounds in the extracts from the leaves, but on the basis of literature data [1] it can be concluded that naphthoquinones and flavonoids are considered as major phenolic compounds of* Juglans regia* leaves. Juglone (5-hydroxy-1,4-naphthoquinone) is known as being the characteristic compound of *Juglans* spp. and is reported to occur in fresh walnut. As dried leaves were used, juglone was not detected in any extract. Furthermore, several hydroxycinnamic acids (3-caffeoylquinic, 3-*ρ*-coumaroylquinic and 4-*ρ*-coumaroylquinic acids), and flavonoids (quercetin 3-galactoside, quercetin 3-arabinoside, quercetin 3-xyloside, quercetin 3-rhamnoside, and two other partially identified quercetin 3-pentoside and kaempferol 3-pentoside derivatives) of different walnut cultivars collected at different times were studied by other research groups in a previous work [19].

As with the qualitative profile, extracts from *Juglans regia *fruits from both stages of maturity contained the same phenolic compounds. However, as further results presented in Table 2, their content was significantly different.

### 3.2. Phenolic Content

As Table 2 presents, all parts of *J. regia *contained significant amount ethanol-extractable phenolic compounds (including flavonoids and phenolic acids). Taking into account total phenolics it was observed that the highest amount contained fruits in both maturity stages (111.31 ± 5.38 and 100.25 ± 4.31 mg/g FM for F3 and F25, resp.). In turn, the highest content of flavonoids was observed in husks (164.61 ± 8.23 mg/g FM). However, the lowest amount of flavonoids (16.47 ± 0.65 mg/g FM) was determined in fruits at the youngest stage of maturity. The highest concentration of phenolic acids (321.81 ± 16.35 *μ*g/g FM) was found for walnut fruits at the youngest stage of maturity while the lowest was found in the leaves of *J. regia.*

Consumption of certain phenolics in the food is considered beneficial for human nutrition [20]. Epidemiological evidence shows that foods rich in phenolics derived from fruits is associated with lower risks of cancer and coronary heart disease, as well as cataracts, brain and immune dysfunction, and stroke [21]. Phenolics also contribute to astringent but pleasant taste of the liqueur [2]. Alamprese et al. [22] studied the antioxidative potential of walnut liqueur and proved that antioxidant activity was directly correlated with the total phenol content and this characteristic did not change during storage, even for many years. Halvorsen et al. [23] reported that walnuts have one of the highest contents of antioxidants among all analysed nuts and seeds. The phenolic content was influenced by ripeness of fruits. Temperature and length of steeping of the fruits in ethanol have little effect on the phenolic composition of the liqueur. Jakopic et al. [24] in their work analyzed how much cultivar and maturation stage of the walnut influence the phenolic content in various parts of the walnut and in traditionally prepared walnut liqueur. These results indicate that early stage of maturity of walnut harvested on June 30 contained higher amounts of phenolic compounds than fruit harvested at a later stage, on July 7. Similar results were obtained in the present study. Chemical extracts prepared with walnuts collected on July 3 were characterized by higher content of phenolic compounds than tinctures obtained from nuts harvested on July 25. Furthermore, Stampar et al. [2] observed variation of certain phenolic compounds in green walnut husks during the growing season, and they ascertained that the majority of the phenolics under investigation decreased during maturation, and similar results were obtained in this study.

### 3.3. Antioxidant Potential of *J. regia* Extracts

It has been recognized that phenolic compounds are a class of antioxidant agents, which act as free radical terminators. Free radicals are involved in many disorders, such as neurodegenerative diseases, cancer, and AIDS. Antioxidants, through their scavenging power, are useful for the management of those diseases. The mechanisms of action of flavonoids are through scavenging or chelating processes [25–27]. The antioxidant capacity of walnut polyphenols has already been described. Anderson et al. [28] reported the *in vitro* inhibition of human plasma and low density lipoprotein (LDL) oxidation by a walnut extract containing ellagic acid, gallic acid, and flavonoids. Flavonoids can also protect cells by acting as free radical scavengers, inhibiting DNA damage and mutagenicity [29].

**Figure 2:**
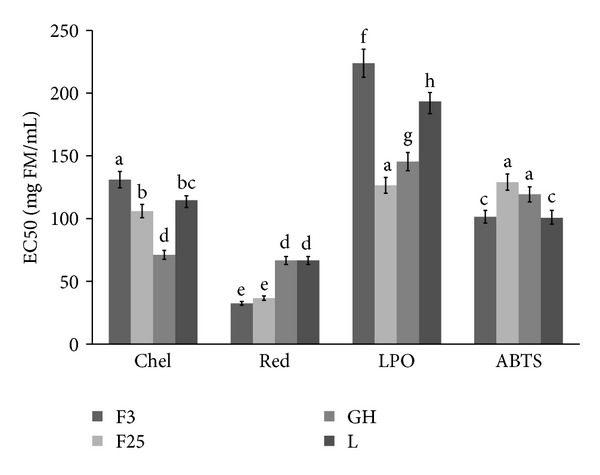
Antioxidant activities of tinctures from different *J. regia* parts. CHEL: chelating power, RED: reducing power, LPO: inhibition of lipid peroxidation, ABTS: antiradical activity, F3: extract from walnut harvested 3.07.2012, F25: extract from walnut harvested 25.07.2012, GH: extract from green husks of walnut, and L: extract from walnut leaves. Bars (means) followed by the different letters differ significantly (Tukey's test, *P* < 0.05).

In the present work, the antioxidant potential of ethanolic extracts of *J. regia* fruits, green husks, and leaves was measured by four different assays: chelating power, reducing power, inhibition of lipid peroxidation, and scavenging activity on ABTS radicals (Figure 2).

The highest ability to chelate Fe^2+^ was observed for tinctures from GH (EC_50_ = 71.01 ± 3.55 mg FM/mL), whereas the lowest was observed (EC_50_ = 131.06 ± 6.55 mg FM/mL) for tincture from F3. All tested parts of *J. regia* contained ethanol-extractable compounds with high reducing power. The highest activity was determined for tinctures from F3 and F25 (EC_50_ = 32.47 ± 1.53 and 36.07 ± 1.72 mg FM/mL, resp.), while activity of other samples were significantly lower (EC_50_ value averaged about 66 mg FM/mL). Contrary to previous results, ability of tested tinctures to prevent lipids against oxidation was relatively low. The highest EC_50_ values were observed for F3 and L samples (223.81 ± 11.19 and 193.27 ± 9.66 mg FM/mL, resp.). The highest activity (EC_50_ = 126.49 ± 6.32 mg FM/mL) was determined for F25 sample. Tested tinctures showed relatively high antiradical activity—EC_50_ values ranged from 100.56 ± 5.03 to 129.04 ± 6.45 mg FM/mL for L and F25, respectively. These results suggest that walnuts collected at early stage of maturity and leaves were the best ABTS radicals scavengers (Figure 2).

In several reports, the antioxidant activity of *J. regia* has been described, especially from walnut oil extracts [30, 31] and traditional walnut liqueurs [2]. Furthermore, Pereira et al. [1] reported the antioxidant potential of walnut leaves. Antioxidant activity was accessed by the reducing power assay and the scavenging effect on DPPH (2,2-diphenyl-1-picrylhydrazyl) radicals. In a general way, all of the studied walnut leaves cultivars presented high antioxidant activity (EC_50_ values lower than 1 mg/mL). The main mode of action of natural antioxidants is their ability to scavenge free radicals before they can initiate free radical chain reactions in cellular membranes or lipid-rich matrices, as found in cosmetics, foodstuffs, and pharmaceutical preparations [32].

A significant role in catalysis of oxidative processes leading to the formation of hydroxyl and peroxyl radicals in the Fenton reaction (O_2_^•−^ + H_2_O_2_→ •OH + HO^−^ + O_2_) plays presence of transition metal ions. These processes can be delayed and inactivation by chelating iron ions [26, 27]. Furthermore, lipid peroxides and hydrogen peroxide, in the presence of the transition metals initiate the chain reaction of lipid peroxidation that continues until it is interrupted by an antioxidant [33]. Iron salts in a biological system attach to biological molecules, where they cause site-specific formation of •OH radicals and consequent damage to lipid, protein, and DNA. Propagation reactions of lipid peroxidation in a biological membrane do not proceed far before they reach a protein; thus, lipid peroxidation *in vivo* causes substantial damage to membrane proteins [34]. Almeida et al. [35] in their studies have shown that aqueous extracts of the leaves of walnut can be a source easily accessible natural antioxidants. In addition, they showed that tested extract may be helpful in the prevention of lipid peroxidation present in food. In addition, the antioxidant activity of the extract of *J. regia *leaves were justified for the therapeutic use in inflammatory conditions. On the other hand, studies conducted by Amaral et al. [19] demonstrated that a high content of tocopherol and vitamin E in walnut prevent oxidation of lipids. Although, Foti et al. [36] showed that flavonoids are a group of compounds which are most active in inhibiting peroxidation of linoleic acid.

### 3.4. Effect on the Activity of Some Enzymes from the Class of Oxidoreductases

Research on dietary polyphenols has intensified over the past decade, mainly due to the direct radical scavenging properties of many such compounds. More recently, however, it has become evident that polyphenols may also decrease oxidative stress through indirect antioxidant action, such as the inhibition of ROS-producing enzymes such as lipoxygenase (LOX) and xanthine oxidase (XO) [37].

Xanthine oxidase (EC 1.1.3.22) is a member of the xanthine oxidoreductase (XOR) group, found in mammals at the highest concentration within the liver and intestine. Among several mechanisms, XO may be a potential source of superoxide and hydrogen peroxide. The predominant xanthine dehydrogenase (XDH) form can be converted into XO under severe conditions, such as ischemic injury, and thereby cause increased oxidative stress [35]. Both XDH and XO convert hypoxanthine to uric acid via xanthine. Excessive levels of uric acid *in vivo* may also lead to a state of hyperuricemia andrenal stones. Various studies have also associated the involvement of XO with thermal stress, respiratory syndrome, viral infection, and hemorrhagic shock. It could therefore be hypothesized that a decreased activity of XO may be considered to be beneficial to health [37, 38].

Lipoxygenase (EC. 1.13.11.12, linoleate: oxygen oxidoreductase) catalyzes the oxygenation of polyunsaturated fatty acids containing a *cis,*-*cis-*1,4-pentadiene system to hydroperoxides. The lipoxygenase pathway of the arachidonic acid metabolism produces ROS and these reactive forms of oxygen and other arachidonic acid metabolites might play a role in inflammation and tumor promotion. Inhibition of the arachidonic acid metabolism is also correlated with tumor promotion in animal models [27, 39]. The interaction of flavonoids with mammalian 15-LOX-1 merits particular attention, as this enzyme is a potential target for the health-preserving effect of flavonoids [40].

**Figure 3:**
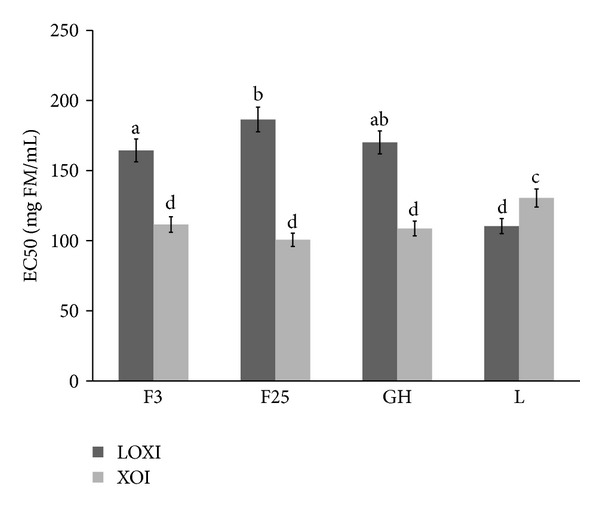
Comparison of LOX and XO inhibitory activity of tinctures from different *J. regia* parts. F3: extract from walnut harvested 3.07.2012, F25: extract from walnut harvested 25.07.2012, GH: extract from green husks of walnut, and L: extract from walnut leaves. Bars (means) followed by the different letters differ significantly (Tukey's test, *P* < 0.05).

As Figure 3 shows, all kinds of extracts were a good source of LOX and XO inhibitors. The highest LOX inhibition was observed for the extract from the leaves of walnut (EC_50_ = 110.45 mg FM/mL). Other extracts showed a similar activity against LOX, and EC_50_ value was from 164.42 mg FM/mL for F3 extract to 186.42 mg FM/mL for F25 extract. On the other hand, extract from *J. regia* fruits at second stage of maturity (F25) and extract from green husks were the best XO inhibitors: EC_50_ = 100.66 mg FM/mL and 108.69 mg FM/mL, respectively. Slightly higher EC_50_ values were noted for the two other extracts.

In the available literature there are no data on the effect of walnut extract on the activity of LOX and XO. Therefore, the results indicating the ability of walnuts and tinctures derived from a variety of vegetative parts to inhibition of these prooxidant enzymes should be emphasized which shows that walnuts and tinctures derived from a variety of vegetative parts possess an ability to inhibit these prooxidant enzymes (Figure 3).

Dew et al. [37] in their work studied the dietary role of XO inhibitors from different varieties of teas, herbs, fruit juices, vegetables, and fruits. They found that a particularly high potential inhibitory activity of XO showed cranberry juice and dark grape. Furthermore, Keßler et al. [25] demonstrated and reported cranberry juice as a protective agent against urinary tract infection and kidney stone prevention product. Saruwatari et al. [41] have shown that drinking twice a day extract prepared from 2.5 g of a herb (ginseng and ginger) for 5 days inhibited by 20–25% of the activity of XO. On the other hand, Dew et al. [37] in their study compared the black, white and herbal teas. The highest inhibitory activity XO had black tea, closely followed by mint tea. They also showed that caffeine has no effect on the decrease or increase in the activity of XO.

Most living organisms possess efficient enzymatic and nonenzymatic defense systems against the excess production of ROS. Antioxidant enzymes, in particular superoxide dismutase (SOD) and catalase (CAT), are involved in cell defense mechanisms against oxidative damage. Antioxidant enzymes have an enormous theoretical advantage over exogenous antioxidants that are stoichiometrically consumed [42].

Catalase is a fundamental defense enzyme which catalyzes the dismutation reactions of hydrogen peroxide, which is one of the ROS.

**Figure 4:**
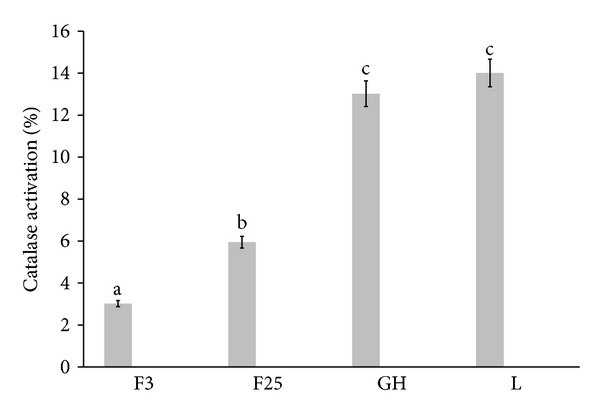
Comparison of CAT-activatory abilities of tinctures from different *J. regia* parts. F3: extract from walnut harvested 3.07.2012, F25: extract from walnut harvested 25.07.2012, GH: extract from green husks of walnut, and L: extract from walnut leaves. Bars (means) followed by the different letters differ significantly (Tukey's test, *P* < 0.05).

In the present study, the ability of extracts to increase CAT activity was investigated (Figure 4). It was observed that best CAT activators were tinctures from the leaves and walnut green husks, increasing CAT activity turn notify 14.01% and 13.02%, slightly weaker activity was observed for tinctures of *J. regia* fruits at both stages of maturity.

According to the free radical theory of ageing, one might expect the activity of antioxidant enzymes to be altered. The activity of these enzymes has been reported to either increase or decrease during the ageing process. Guemouri et al. [43] reported that CAT activity decreased in >65-year-old French population. On the other hand, Inal et al. [44] found that CAT activity increased with ageing. The increase in CAT activities with age suggests an increase in H_2_O_2_ formation. During the ageing process steady-state concentrations of erythrocyte H_2_O_2_ might be considerably higher, which could lead to the induction of antioxidative enzymes, an adaptive phenomenon. For this reason, the consumption of food rich in CAT activators, such as walnut tincture, might be effective in preventing the pathophysiological changes of ageing.

## 4. Conclusion

Undoubtedly, walnuts and ethanol extracts prepared from different vegetative parts are a rich source of phenolic compounds that has been proven by many researchers working on this theme. Furthermore, these compounds can enhance the effect of other antioxidants, such as fat-soluble vitamins and low molecular water soluble substances. Moreover, the high content of antioxidant components in plants, decide on their significant role in the prevention of lifestyle diseases. The results obtained suggest that *J. regia* can be a source of bioactive compounds with antioxidant properties. On the basis of the analysis it can be concluded that the fruits of walnut in the early stages of maturity contain significantly more biologically active compounds than in the later stages of fruit maturity. Particularly noteworthy is the activity of the compounds contained in the leaves of *Juglans regia*, which may be easily accessible source of valuable substances.

## Figures and Tables

**Table 1:** Identification and occurrence of major phenolic compounds in *Juglans regia* fruits (F3, F25), green husks (GH), and leaves (L) tinctures. Elution times correspond to the separation by UPLC. Molecular mass was determined by MS as *m*/*z*. Compounds were fragmented in MS/MS experiments and the *m*/*z* values for the main daughter ions are given in brackets.

No.	Elution time (min)	[M-H]^−^ ion, MS^2^ daughter ions	UV absorbance peaks (nm)	Compound identity	L	F3	F25	GH
1	1.09	169 (125)	271	Gallic acid	+	+	+	+
2	1.99	153 (109)	259, 293	Protocatechuic acid	+	+	+	+
3	2.47	353 (191, 179)	324	3-Caffeoylquinic acid	+	+	+	+
4	2.97	181	297	Unknown	+	+	+	+
5	3.16	341 (281, 179)	325	Caffeic acid hexoside I	+	−	−	−
6	3.40	337 (163)	310	3-*p*-Coumaroylquinic acid	+	+	+	+
7	3.64	341 (281, 179)	323	Caffeic acid hexoside II	+	−	−	+
8	4.06	353 (179,173)	325	4-Caffeoylquinic acid	+	+	+	+
9	4.26	451 (405)	310	Unknown	+	−	−	−
10	4.60	339 (159)	258, 313	Unknown	+	+	+	+
11	4.76	325 (265, 163)	310	*p*-Coumaric acid hexoside	+	−	−	−
12	4.88	177 (159, 115)	259, 317	Unknown	−	+	+	+
13	5.14	337 (173)	311	4-*p*-Coumaroylquinic acid	+	+	+	+
14	5.29	281	267	Unknown	−	−	−	+
15	5.60	163 (119)	308	*p*-Coumaric acid	+	+	+	+
16	5.67	193 (175)	260, 364	Unknown	−	+	+	+
17	5.86	513 (453, 409, 289)	260, 364	Unknown	−	+	+	−
18	5.99	197 (169, 125)	273	Unknown	−	+	+	−
19	6.38	435 (285, 151)	290	Unknown	+	−	−	−
20	6.79	381 (161)	253, 360	Unknown	−	+	+	−
21	6.84	435 (303, 285, 151)	290	Unknown	+	−	−	+
22	7.24	463 (301)	255, 353	Quercetin-3-*O*-hexoside I	+	−	−	+
23	7.41	491 (331, 271)	264	Unknown	−	+	+	+
24	7.47	463 (301)	255, 352	Quercetin-3-*O*-hexoside II	+	−	−	−
25	7.57	435 (303, 285, 151)	290	Unknown	+	+	+	+
26	7.83	433 (301)	255, 352	Quercetin-3-*O*-pentoside I	+	−	−	−
27	8.02	433 (301)	255, 354	Quercetin-3-*O*-pentoside II	+	−	−	−
28	8.11	447 (285)	264, 350	Kaempferol-3-*O*-hexoside	+	−	−	−
29	8.28	433 (301)	256, 352	Quercetin-3-*O*-pentoside III	+	−	−	−
30	8.61	447 (301)	255, 346	Quercetin-3-*O*-deoxyhexoside	+	+	+	+
31	8.86	417 (285)	265, 346	Kaempferol-3-*O*-pentoside I	+	−	−	−
32	9.05	417 (285)	265, 346	Kaempferol-3-*O*-pentoside II	+	−	−	−
33	9.24	477 (285, 151)	290	Unknown	+	−	−	−
34	9.41	417 (285)	364, 345	Kaempferol-3-*O*-pentoside III	+	−	−	−
35	9.84	431 (285)	264	Kaempferol deoxyhexoside	+	−	−	−
36	10.37	501 (281, 179)	324	Dicaffeic acid hexoside	+	−	−	−
37	10.51	489 (301)	252, 337	Quercetin-3-*O*-acetyl-deoxyhexoside I	+	−	−	−
38	10.57	475 (301)	254, 348	Quercetin-3-*O*-acetyl-pentoside	+	−	−	−
39	11.00	609 (463, 301)	263	Quercetin deoxyhexoside-hexoside	+	−	−	−
40	11.30	489 (301)	255, 361	Quercetin-3-*O*-acetyl-deoxyhexoside II	+	−	−	−
41	11.68	485 (265, 163)	262, 312	Unknown	+	−	−	−
42	11.84	473 (285)	265, 312	Kaempferol acetyl-deoxyhexoside I	+	−	−	−
43	12.34	469 (145)	300	Unknown	+	−	−	−
44	12.49	473 (285)	264, 311	Kaempferol acetyl-deoxyhexoside II	+	−	−	−
45	12.72	473 (285)	262	Kaempferol acetyl-deoxyhexoside III	+	−	−	−
46	13.32	285	265, 363	Kaempferol	+	−	−	−

Plus (+) and minus (−) signs represent occurrence of each compound.

**Table 2:** Comparison of total phenolics, total flavonoids, and phenolic acids content in different parts of *J. regia. *

Plant material	Total phenolic content [mg/g FM]	Total flavonoids content [mg/g FM ]	Total phenolic acids content [*μ*g/g FM ]
F3	111.31 ± 5.38^a^	10.22 ± 0.55^a^	321.81 ± 16.35^a^
F25	100.25 ± 4.36^b^	113.56 ± 5.32^b^	284.65 ± 12.54^b^
GH	52.48 ± 1.23^c^	164.61 ± 8.23^c^	309.45 ± 10.26^c^
L	6.72 ± 0.35^d^	16.47 ± 0.65^d^	26.14 ± 4.78^d^

F3: extract from walnut harvested 3.07.2012, F25: extract from walnut harvested 25.07.2012, GH: extract from green husks of walnut, and L: extract from walnut leaves.

Bars (means) in columns followed by the different letters differ significantly (Tukey's test, *P* < 0.05).
